# Supplementary material for: Degrees of compositional shift in tree communities vary along a gradient of temperature change rates over one decade: Application of an individual‐based temporal beta‐diversity concept
Source: Ecol Evol. 2020 Sep 24;10(24):13613–23. doi: 10.1002/ece3.6579 (PMC7771126; doi:10.1002/ece3.6579)
Supplement: Supplementary file 1 — Appendix S1 [file ECE3-10-13613-s001.zip › ece36579-sup-0001-AppendixS1/ece36579-sup-0003-TextS1.docx]

**Supplementary text 1 Explanation of individual-based temporal beta-diversity in the Ružička dissimilarity index version**

In the main text, I explained only an extension of the Bray–Curtis dissimilarity index. Here, the case of individual-based temporal beta-diversity based on the Ružička dissimilarity index is presented. Specifically, the widely used Ružička dissimilarity is considered an abundance-based extension of the Jaccard dissimilarity index. The following equations refer to equations 4, 8, 9, 13, 14, 15, and 16 in the main text that are related to the Bray–Curtis dissimilarity.

$d_{Ruz}= \frac{B+C}{A+B+C}$. (18)

$d_{t.Ruz}= \frac{B_{t}+C_{t}}{A_{t}+B_{t}+C_{t}}$. (19)

$d_{t.Ruz}= \frac{B_{t}+C_{t}}{A_{t}+B_{t}+C_{t}}$

$= \frac{B_{t}}{A_{t}+B_{t}+C_{t}}+\frac{C_{t}}{A_{t}+B_{t}+C_{t}}=d_{t.Ruz.loss}+d_{t.Ruz.gain}$. (20)

$d_{Ruz.MR}= \frac{M+R}{P+M+R}$. (21)

$d_{Ruz.MR}= \frac{M+R}{P+M+R}$

$=\frac{M}{P+M+R}+\frac{R}{P+M+R}=d_{Ruz.M}+d_{Ruz.R}$. (22)

$d_{MR}= \frac{M+R}{P+M+R}$

$= \frac{B_{t}+E_{loss}+C_{t}+E_{gain}}{P+M+R}= \frac{B_{t}+C_{t}+2E}{P+M+R}$

$= \frac{2E}{P+M+R}+\frac{B_{t}+C_{t}}{P+M+R}=d_{Ruz.E}+ d_{Ruz.S}$ (23)

$v_{Ruz.s}= \frac{B_{t}+C_{t}}{E+B_{t}+C_{t}}$

$=1-\frac{E}{E+B_{t}+C_{t}}=1-v_{Ruz.e}$. (24)
