# Supplementary figures and images for: Degrees of compositional shift in tree communities vary along a gradient of temperature change rates over one decade: Application of an individual‐based temporal beta‐diversity concept
Source: Ecol Evol. 2020 Sep 24;10(24):13613–23. doi: 10.1002/ece3.6579 (PMC7771126; doi:10.1002/ece3.6579)

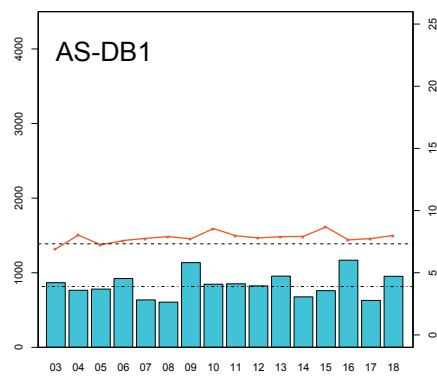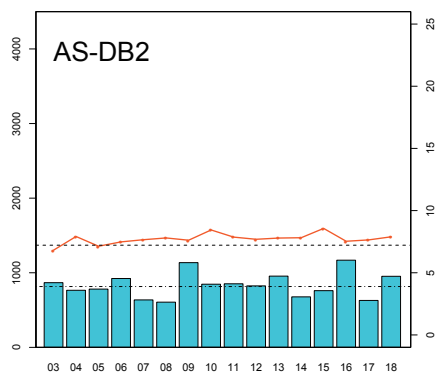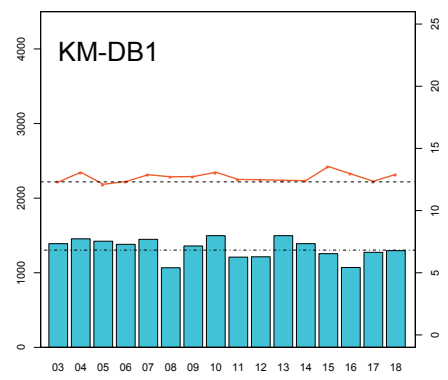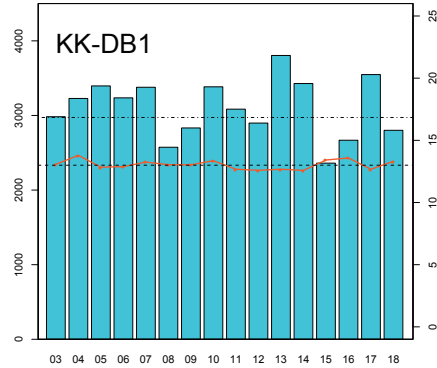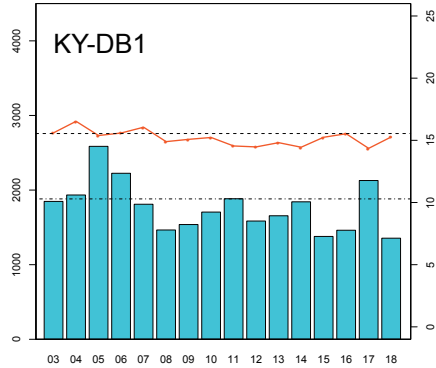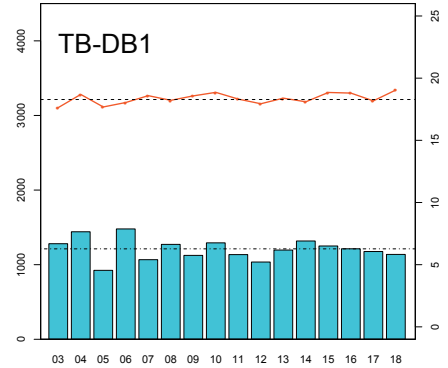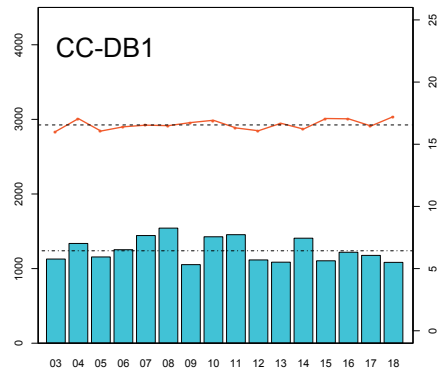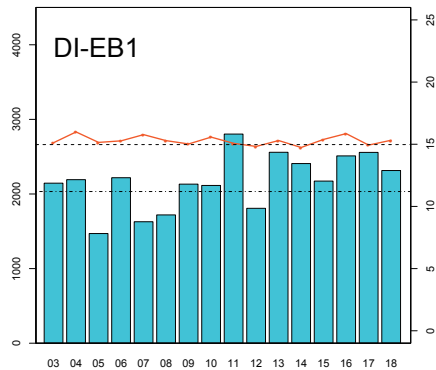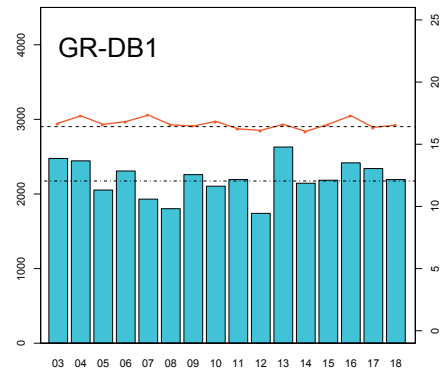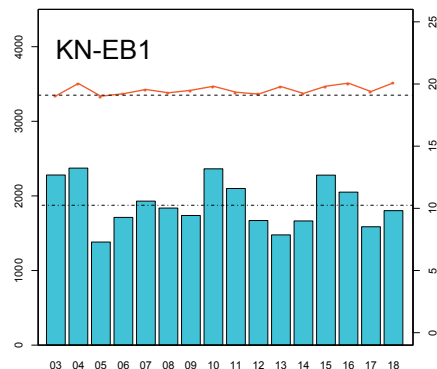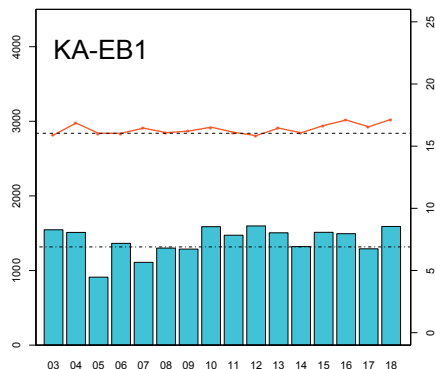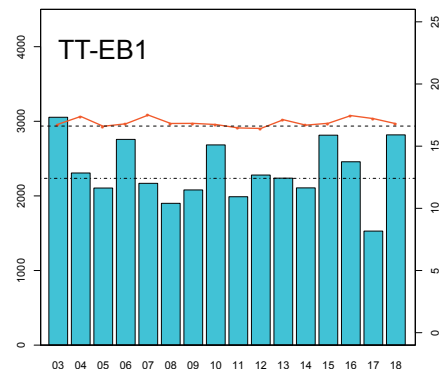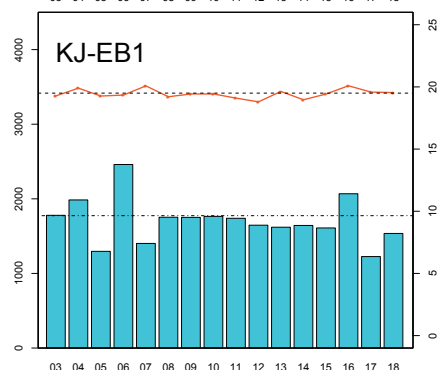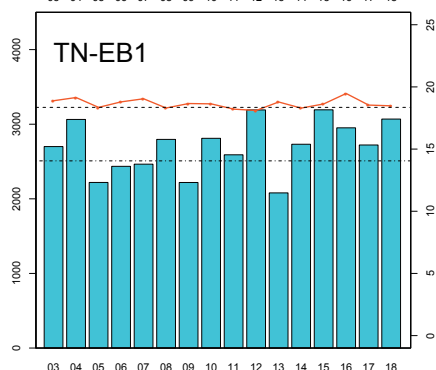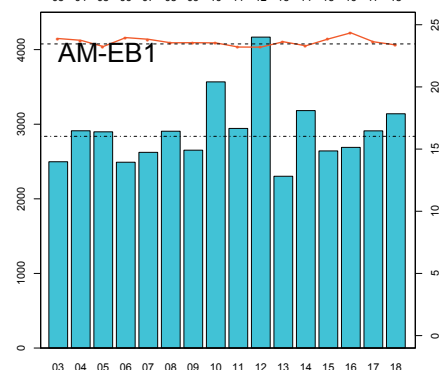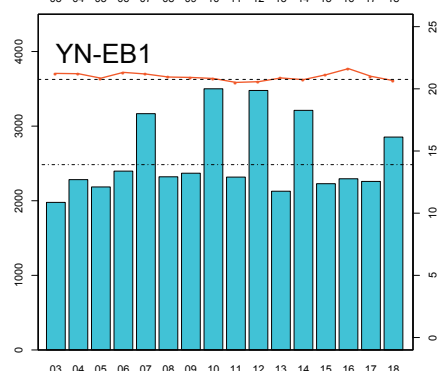

Supplement: Supplementary file 1 — Appendix S1 [file ECE3-10-13613-s001.zip › ece36579-sup-0001-AppendixS1/ece36579-sup-0001-FigS1.pdf]
